# Integrin α3β1 Is Not Required for Onset of Dysplasia in Genetic Model of Colon Cancer but Promotes Motility of Colon Cancer Cells

**DOI:** 10.3390/cancers17030371

**Published:** 2025-01-23

**Authors:** Kathryn E. Ottaviano, Sita Subbaram, Lei Wu, Kiley Stahl, Antoinette J. Mastrangelo, Hwajeong Lee, C. Michael DiPersio

**Affiliations:** 1 Department of Surgery, Albany Medical College, Albany, NY 12208, USA; ottavik@amc.edu (K.E.O.); subbarsi@amc.edu (S.S.); wul2@amc.edu (L.W.); 2 Department of Molecular & Cellular Physiology, Albany Medical College, Albany, NY 12208, USA; kstahl@uri.edu (K.S.); mastraa1@amc.edu (A.J.M.); 3 Department of Biology, Union College, Schenectady, NY 12308, USA; 4 Department of Pathology, Albany Medical College, Albany, NY 12208, USA; leeh5@amc.edu

**Keywords:** integrin α3β1, colorectal cancer, colonic dysplasia, KPC:APC mouse model

## Abstract

Background/Objectives: The progression of colorectal cancer through clinically and histopathologically well-defined stages is driven by specific mutations that activate oncogenes or inactivate tumor-suppressor genes. In addition, pre-cancerous/cancer cells respond to cues from the tissue microenvironment that support tumorigenesis and progression, many of which are transmitted through integrin receptors for the extracellular matrix. Integrin α3β1 has pro-tumorigenic/pro-metastatic roles in many cancers, but it also has suppressive roles in some cancers or at specific stages of progression, indicating that its potential value as a therapeutic target cannot be extrapolated across cancer types or stages. In this study, we investigated roles for α3β1 in colorectal cancer using cellular and genetic models that represent different stages. Methods: We generated mice with colon-specific α3 knockout in a tamoxifen-inducible model of *KRAS*-mutated colorectal cancer to assess the effects of α3β1 ablation on early dysplasia. We also used siRNA to suppress α3β1 in human colorectal cancer cells, then assessed effects on motility and invasion in vitro. Results: Genetic deletion of α3β1 in the colon did not alter dysplasia in mice predisposed to *KRAS*-mutated colorectal cancer, and it was accompanied by an increase in the colocalization of α6 integrin with laminin-332 (a matrix ligand for both integrins), suggesting functional compensation. However, suppression of α3β1 caused an approximately 40% to 60% reduction in the motility/invasion of human colorectal cancer cells. Conclusions: Our findings that α3β1 is not required for pre-cancerous dysplasia but promotes colorectal cancer cell motility/invasion indicate an important role for pro-migratory functions of this integrin at later stages of progression when cells invade from the primary tumor, suggesting that strategies to target α3β1 in colorectal cancer should be aimed at distinct stages of disease progression.

## Simple Summary

Integrin α3β1 is an extracellular matrix receptor with pro-tumorigenic/pro-metastatic roles on many tumor/cancer cells, and its expression has been correlated with poor prognosis, indicating its potential value as a therapeutic target and/or prognostic marker. However, α3β1 has also been linked to tumor-suppressive roles in some cancer types/subtypes, and it may even switch from tumor-supportive to cancer-suppressive during disease progression, which highlights the need to investigate this integrin in different cancers and at different stages. In the current study, we assessed roles for α3β1 in colorectal cancer, where its value as a therapeutic target is underexplored. Using a genetic approach to delete α3β1 in a murine model of colorectal cancer, we showed that it is dispensable for pre-cancerous colonic dysplasia. However, using RNAi to suppress α3β1, we showed that it promotes the motility/invasion of human colorectal cancer cells. These findings indicate that α3β1 may be important at specific stages of colorectal cancer progression.

## 1. Introduction

Cancer remains a major global health burden with >19 million new diagnoses and ~10 million cancer deaths estimated in 2020, and this burden is projected to rise almost 50% by 2040 [1]. This burden is also reflected in U.S. statistics, with more than 600,000 cancer deaths and 2 million new diagnoses estimated in 2024 [2]. Although cancer mortality has declined overall—due, in part, to improved detection methods and therapies—during 2015–2019, the incidence of several common cancers increased annually in the U.S. by 0.6–3% including breast, pancreatic, uterine, prostate, and colorectal cancers, and melanoma [2], highlighting a critical need to develop more effective methods for early detection and treatment. Cancers are typically treated through a combination of surgery, radiation therapy and/or chemotherapy, as reviewed [3]. Surgery remains an important treatment as advances in imaging and the detection of circulating tumor-derived DNA continue to improve early detection. While chemotherapy and radiation therapies are often effective, both can lead to undesirable side effects as they also damage healthy cells. Targeted delivery offers a promising strategy to deliver inhibitory drugs or agents directly to cancer cells while minimizing damage to healthy cells, but there remains a need to identify targetable elements that drive tumor development and progression [3].

Colorectal cancer (CRC) is one of the most common cancers worldwide and a leading cause of cancer-related deaths in the U.S. Over the past two decades, CRC incidence has increased in people under the age of 50, and it is now the first (among men) or second (among women) leading cause of cancer death in people of this age group [1,2,4,5]. While the 5-year relative survival rate for localized CRC is ~91%, survival drops sharply to ~16% in patients with cancer that has metastasized to distant parts of the body [6]. As the majority of CRC patients are diagnosed with locally advanced or disseminated disease [7], there is an urgent need for new strategies to detect and treat CRC in the clinic.

Distinct stages of CRC are associated with specific genetic mutations in colonic epithelial cells that drive progression to malignant disease [4,8]. A common initiating event is loss-of-function mutation in the tumor suppressor, adenomatous polyposis coli (APC), which leads to disruption of the Wnt signaling pathway and dysregulated cell growth that promotes the formation of intestinal polyps. Subsequent gain-of-function mutations in proto-oncogenes, together with loss-of-function mutations in other tumor-suppressor genes, drive the multistep progression to carcinoma [4,9,10]. *KRAS* gene mutations are correlated with tumor aggressiveness and poor prognosis, and they are acquired in 40–45% of CRC patients, mainly at the transition from low- to high-grade dysplasia [11,12]. It is estimated that ~25% of patients with sporadic CRC carry both *APC* and *KRAS* mutations [13].

In addition to driver mutations, changes that occur in the tumor microenvironment (TME) are critically important to support tumor growth and promote malignant progression [7,14]. Integrins are cell-surface receptors for the extracellular matrix (ECM) that consist of an α and a β subunit and facilitate bidirectional signaling across the cell membrane [15,16]. A number of integrins have important regulatory roles in both tumor cell-intrinsic functions (e.g., proliferation, survival, invasion) and the tumor cell-mediated modification of the TME that supports cancer development and progression [17]. The laminin-binding integrin α3β1 is expressed in the epithelial cells of intestine and other tissues [18,19], and it has pro-tumorigenic/pro-metastatic roles in many cancer types [20,21,22,23,24,25,26,27]. Indeed, α3β1 has been shown to promote the motility of several tumor cell types in in vitro migration assays, which may reflect a role in the local invasion from the primary tumor that precedes metastasis [21,28,29,30,31,32]. However, α3β1 has also been shown to have suppressive roles in tumorigenesis or the metastasis of certain cancers or cancer subtypes [22,25,33,34]. Moreover, α3β1 can have opposing effects at early versus late stages of some cancers [25]. For example, absence of α3β1 leads to the reduced initiation and growth of epidermal tumors [27,35], but α3β1-deficient tumors show an increased rate of progression to invasive carcinomas [35], which highlights the contrasting roles for this integrin at different stages of squamous cell carcinoma (SCC) development and suggests that functions of α3β1 are dynamically regulated during disease progression. Collectively, the above findings highlight the need to investigate integrin α3β1 in each type of cancer, as well as at different stages of progression within a cancer type, when considering its value as a potential therapeutic target or prognostic marker.

While it is clear that α3β1 plays important and sometimes contradictory roles in several cancers, its role in CRC is underexplored. Some studies have reported that suppressing or inhibiting α3β1 in colon carcinoma cells reduces their proliferation, invasion and/or migration [30,31], and activated *KRAS* has been linked to increased α3β1 expression in colon carcinoma cells [36], suggesting cancer-supportive roles for this integrin. On the other hand, lower expression of the *ITGA3* gene (which encodes the α3 integrin subunit) has been correlated with metastasis and poor prognosis in CRC patients [37,38,39], and α3β1 expression was reported to be reduced during the transition of colon tumors from benign to malignant [40]. These disparate findings highlight the need to investigate requirements for α3β1 at specific stages of CRC development.

In the current study, we crossed mice with a floxed *Itga3* gene [41] into the tamoxifen-inducible KPC:APC mouse model of *KRAS*-mutated CRC [13], thereby generating mice with a tamoxifen-inducible “molecular switch” to simultaneously knockout α3 in the colon and activate two driver mutations (i.e., loss of *Apc* and activation of oncogenic *Kras*). We observed that α3 knockout did not alter the histology of normal colon in our experimental timeframe, nor did it alter the colonic dysplasia that occurs in tamoxifen-treated KPC:APC mice, indicating that α3β1 is not essential for pre-cancerous dysplastic growth. Interestingly, immunohistology showed that the deletion of α3 in either the normal or dysplastic colon was associated with the enhanced colocalization of α6 integrin and laminin-332 (LN-332) (an ECM ligand shared between α3β1 and α6β4), possibly reflecting functional compensation by α6β4 in the absence of α3β1. Since KPC:APC mice generated through this intercross succumbed to cachexia before they could form tumors, we used RNAi to knockdown *ITGA3* in two human CRC cell lines, SW480 and HCT116. Transwell migration/invasion assays showed that the suppression of α3β1 caused reduced motility in both cell lines. Our findings that α3β1 is pro-migratory/pro-invasive in CRC cells but dispensable for colonic dysplasia during early stages of *KRAS*-mutated CRC indicate stage-specific requirements for this integrin during CRC progression, and they raise important considerations regarding the exploitation of α3β1 as a potential therapeutic target for CRC treatment.

## 2. Materials and Methods

### 2.1. Genetically Engineered Mouse Models

**Figure 1:**
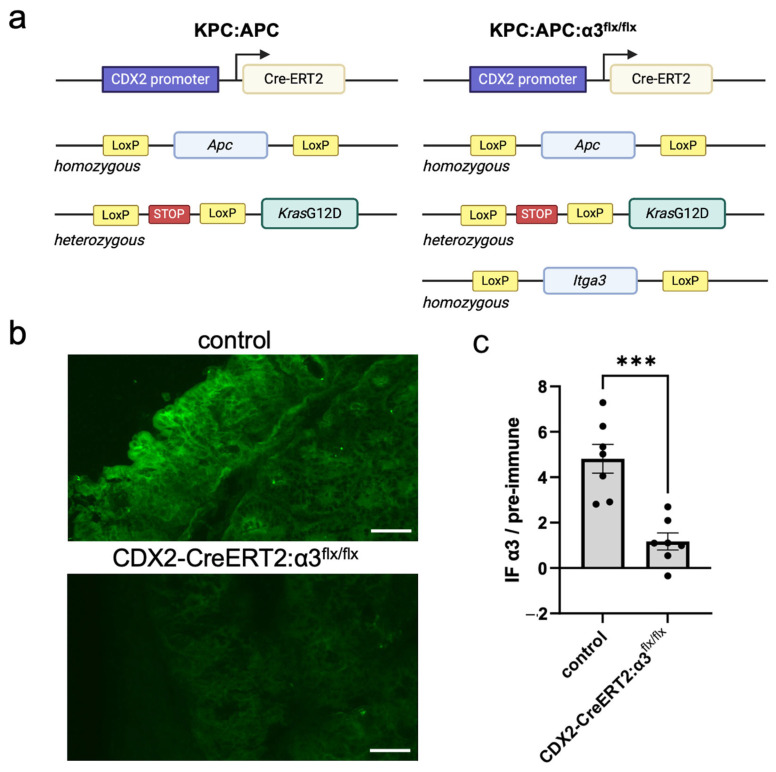
Genetic model for deletion of *Itga3* in a tamoxifen-inducible model of colorectal dysplasia and cancer. (**a**) Schematic of KPC:APC and KPC:APC:α3^flx/flx^ mice; genetic components are described in the text (created with BioRender.com). (**b**) IF staining with anti-α3 of colon cryosections from a control mouse (i.e., lacking Cre) or a CDX2-CreERT2:α3^flx/flx^ mouse, 9 days following the initial IP injection of tamoxifen (see Section 2). Scale bars, 50 μm. (**c**) Quantification of intensity of anti-α3 staining above pre-immune staining in adjacent cryosections (n = 7 for control or CDX2-CreERT2:α3^flx/flx^; mean ± SEM; *** *p* < 0.001; two-tailed *t*-test).

*Itga3*^flx/flx^ mice [41] were intercrossed with KPC:APC mice (Strain #035169; Jackson Laboratories, Bar Harbor, ME, USA) to generate KPC:APC:α3^flx/flx^ mice on a mixed-strain background (see Figure 1a). This intercross strategy also produced CDX2-CreERT:α3^flx/flx^ mice that do not carry the *Kras*G12D oncogene or floxed alleles of *Apc*, such that tamoxifen treatment leads to the colon-specific knockout of α3 in mice that are not susceptible to colorectal dysplasia or tumorigenesis. The activation of *Kras*G12D and deletion of *Apc*^flx^ and *Itga3*^flx^ alleles were achieved by treating mice with two intraperitoneal (IP) injections of tamoxifen 2 days apart (0.05 mg/20 gm body weight in 100 μL corn oil; Caymen Chemical Company, Ann Arbor, MI, USA), as described [13]. When mice lost 20% of their body weight or became moribund (9–14 days after the initial tamoxifen injection), they were euthanized by CO_2_ narcosis. Colons were excised, cut lengthwise, and either embedded in paraffin or frozen in OCT (Electron Microscopy Sciences; Hatfield, PA, USA). Approval for all mouse experiments was granted by Albany Medical College’s Institutional Animal Care and Use Committee.

### 2.2. Histology

For paraffin sections, fixed colon tissue was paraffin-embedded and 5 µm sections were prepared and stained with hematoxylin and eosin (H&E). For immunofluorescence (IF), 10 µm frozen sections were rehydrated (0.02% Tween-20/PBS) for 10 min, fixed (4% paraformaldehyde/PBS), permeabilized (0.4% TritonX-100/PBS), blocked (0.5% BSA, 10% goat serum, 0.1% Tween-20 for 30 min, or 5% milk, 10% heat-inactivated goat serum for 1 h), and then stained with anti-α3 integrin subunit or the corresponding pre-immune serum (1:400; [41]), anti-α6 integrin subunit (1:500; Cat. #MAB1378, Sigma-Aldrich, St. Louis, MO, USA), anti-cytokeratin-8 (K8) (1:250; Cat. #AB53280, AbCam, Cambridge, UK), or anti-LN-332 (1:200; Cat. #AB14509, AbCam). Secondary antibodies (1:250; Molecular Probes/ThermoFisher Scientific, Eugene, OR, USA) were Alexa Fluor 594 goat anti-rat IgG (Cat. #A-11007), Alexa Fluor 594 goat anti-rabbit IgG (Cat. #A-11012), or Alexa Fluor 488 goat anti-rabbit IgG (Cat. #A-11008). In some cases, sections were co-stained with DAPI to mark nuclei. Sections were mounted with ProLong Gold antifade mounting media (Molecular Probes/ThermoFisher Scientific, Eugene, OR, USA) and imaged on a Nikon Eclipse 80 i microscope with a Photometrics Cool Snap ES camera. IF staining intensity produced by anti-α3 serum or pre-immune serum was quantified from adjacent cryosections using ImageJ software, version 1.53t (National Institutes of Health, Bethesda, MD, USA); then, the anti-α3 signal above the background (i.e., pre-immune signal) was compared between control mice (n = 7) and α3-knockout mice (n = 7) using a two-tailed *t*-test.

### 2.3. Cell Culture

SW480, SW620, and HCT116 cells (American Type Culture Collection, ATCC, Manassas, VA, USA) were cultured in Dulbecco’s modified medium (DMEM) (Cat. 10-013-CV, Corning, Waltham, MA, USA) supplemented with 10% fetal bovine serum (Cat. 100–106, Gemini Bioproducts, West Sacramento, CA, USA) and 1% penicillin-streptomycin (Cat. 15140122, Gibco, Waltham, MA, USA) at 37 °C, 5% CO_2_.

### 2.4. Flow Cytometry

Surface α3β1 levels were measured as described [29] on a FACSymphony A3 (Becton Dickinson, Franklin Lakes, NJ, USA) using a monoclonal antibody against the α3 integrin subunit (MAB1952Z, EMD Millipore, Burlington, MA, USA), or normal mouse IgG as a control (sc-2025, Santa Cruz Biotechnology, Dallas, TX, USA), followed by secondary goat anti-mouse IgG (Cat. A865, Invitrogen, Waltham, MA, USA). Data were analyzed using FlowJo software, version 10 (Becton Dickinson).

### 2.5. siRNA Transfection

Cell cultures on six-well plates (70–80% confluent) were transfected with siRNAs using LipofectamineTM 2000 (Cat. 11668-019, Invitrogen) or RNAiMax (Cat. 13778-075, Invitrogen), as we described [29]. Mission siRNA universal negative control #2 (SIC002, Sigma-Aldrich, St. Louis, MO, USA), anti-α3 siRNAs (Cat. SASI_Hs01_00196578, Cat. SASI_Hs01_00196579, Millipore Sigma, St. Louis, MO, USA), or dicer-substrate non-targeting control (Cat. 51-01-14-03, IDT, Coralville, IA, USA) were incubated with Lipofectamine diluted in OptiMEM (Cat. 31985070, Gibco) and transferred into six-well plates and harvested after 48 h. In all experiments, siRNA-mediated knockdown was routinely validated by qPCR for the α3 mRNA transcript.

### 2.6. RNA Isolation and qPCR

RNA was isolated using the RNeasy Plus Mini Kit (Qiagen, Cat. 74134, Hilden, Germany), and RNA quality was assessed on a NanoDrop 1000 Spectrophotometer (Thermofisher, Waltham, MA, USA). cDNA was made with a iScriptTM cDNA Synthesis Kit (Cat. 1708890, Bio-Rad, Hercules, CA, USA). qPCR was performed using SsoAdvancedTM Universal SYBR^®^ Green Supermix (Cat. 172-5270, Bio-Rad) in a Bio-Rad CFX96 Touch thermocycler at 95 °C for 3 min in one cycle; this was followed by 95 °C for 10 s and 55 °C for 30 s, for 39 cycles. The specificity of qPCR was assessed by melt-curve analysis (55 °C to 95 °C, increments of 0.5 °C). Data using α3-specific primers were normalized to the geometric means of three housekeeping genes (PUM1, IPO8, PSMC4). qPCR primers were designed using the IDT PrimerQuest^®^ tool (IDT, Coralville, IA, USA). α3-specific primer sequences were as follows: Fwd-GCAGGTAATCCATGGAGAGAAG, Rev-CCACTAGAAGGTCTGGGTAGAA.

### 2.7. Transwell Migration and Invasion Assays

To assess cell migration and invasion, we performed transwell assays to measure the capacity for cell motility towards a chemo-attractant gradient, as described [42], as these assays are commonly used to assess the motility of different cancer cell types [29,34,43,44]. For migration assays, cells in serum-free growth medium were seeded onto duplicate 24-well transwell filters (8 μM; Cat. 3422, Corning) with 10% serum in the lower chamber as a chemoattractant, and then they were incubated at 37 °C. For a comparison of SW480 and SW620 cells, 2 × 10^5^ cells were seeded and allowed to migrate for 24 h. For siRNA-treated cells, HCT116 cells (5 × 10^4^) were allowed to migrate for 3 h, while SW480 or SW620 cells (2 × 10^5^ for each) were allowed to migrate for 24 h. For invasion assays of siRNA-treated cells, 2 × 10^5^ SW480 cells or 5 × 10^4^ HCT116 cells in growth medium/10% serum were seeded onto duplicate 24-well Matrigel invasion chambers (Cat. 354483, Corning) with 20% serum in the lower chamber. Plates were incubated at 37 °C for 24 h (SW480 cells) or 18 h (HCT116 cells). For both migration assays and invasion assays, non-migrated cells were removed from the top sides of filters using a cotton swab, and then the filter was fixed in methanol and stained with 40, 6-diamidino-2-phenylindole, dihydrochloride (DAPI). Cells were counted from four separate 10× fields per filter using a Nikon eclipse TE2000-U inverted microscope (Nikon Instruments, Inc., Tokyo, Japan), and then they were quantified and averaged from at least three independent experiments.

### 2.8. CCK8 Cell Viability Assay

Transfected SW480 or HCT116 cells (5 × 10^4^) were seeded onto 96-well plates (Day 0) in complete growth medium. On days 1 and 4, quadruplicate wells were assayed using Cell Counting Kit-8 (CCK-8; Dojindo, Kumamoto, Japan) according to the manufacturer’s instructions and quantified on a BioTek Synergy 2 multi-detection microplate reader (Agilent Technologies, Santa Clara, CA, USA).

### 2.9. Statistical Analyses

Regarding the analysis to compare two experimental groups, a two-tailed Student’s *t*-test was used. For analyses comparing more than two groups, one-way ANOVA with the Dunnett multiple comparisons post-test was used. For the migration and invasion assays, data were normalized to the daily mean. *p* < 0.05 is considered significant.

## 3. Results

### 3.1. Development of a Genetic Mouse Model to Delete Itga3 During Early Stages of Kras-Mutated Colorectal Cancer

As discussed above, some studies have shown reduced α3β1 expression during the malignant progression of CRC [40] or have linked lower *ITGA3* expression to poor clinical outcomes [37,38,39]. Consistently, our use of the Human Protein Atlas [45] to assess correlation between *ITGA3* mRNA expression level and patient survival revealed that while high *ITGA3* expression is prognostic of poor outcomes in head and neck SCC, pancreatic cancer, and lung cancer, low *ITGA3* expression is correlated with poor survival in colon cancer (Figure S1). Since the latter trend appears to contrast with some reports that α3β1 supports the proliferation, invasion and/or migration of CRC cells [30,31], we sought to test requirements for this integrin in experimental models that allow for the assessment of different stages of CRC development and progression.

We first sought to determine the effect of ablating α3β1 on the early stages of CRC development in vivo. For these experiments, we utilized the established genetically engineered KPC:APC model in which conditional mutant alleles predispose mice to the development of colonic tumors following treatment with tamoxifen [13]. As illustrated in Figure 1a, KPC:APC mice are homozygous for a floxed *Apc* allele and heterozygous for an oncogenic G12D point mutation engineered into the endogenous *Kras* gene, such that its expression is blocked by a loxP-flanked stop codon. These mice also carry a CDX2-Cre-ERT2 transgene that directs the expression of a tamoxifen-inducible Cre-ERT2 fusion protein to the epithelium of the distal ileum and throughout the colon from the crypt base to the luminal surface [13]. As described previously, tamoxifen-induced activation of Cre-ERT2 leads to the simultaneous activation of the *Kras*G12D oncogene and loss of the floxed *Apc* tumor-suppressor gene, constituting a “molecular switch” that drives the development of colonic dysplasia and cancer development that is genetically and histologically similar to *KRAS*-mutated colorectal cancer in humans [13]. We crossed a floxed *Itga3* allele [41] into the KPC:APC model to generate KPC:APC:α3^flx/flx^ mice, in which tamoxifen treatment (see Section 2) causes the Cre-mediated deletion of α3 simultaneously with the activation of the cancer-causing genetic lesions (Figure 1a). A loss of α3 from the colon of CDX2-CreERT2:α3^flx/flx^ mice following an IP injection of tamoxifen (see Section 2) was confirmed by the IF staining of cryosections with an antiserum specific for α3, which was reduced compared with tamoxifen-treated mice that lack the CDX2-CreERT2 transgene (Figure 1b). Quantification of IF intensity in colon sections collected 9 days after initial tamoxifen treatment showed that anti-α3 staining in CDX2-CreERT2:α3^flx/flx^ mice was reduced to the near-background levels seen in staining with the corresponding pre-immune serum (Figure 1c). Importantly, reduced α3 staining indicates a loss of integrin α3β1, since the α3 subunit pairs exclusively with the β1 subunit [15,19].

### 3.2. Genetic Deletion of Itga3 Does Not Alter Normal Colon Histology

**Figure 2:**
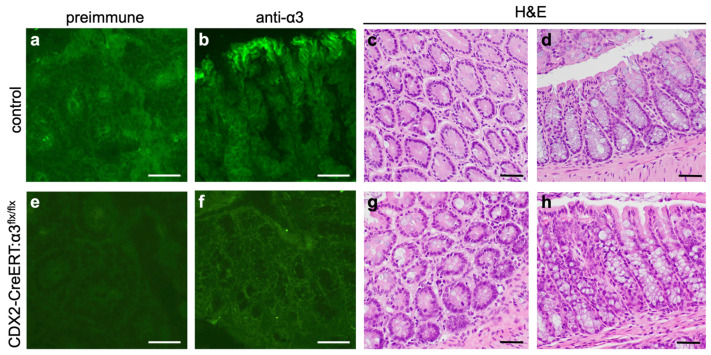
Genetic deletion of *Itga3* does not alter tissue structure of the adult colon. Colon tissue was isolated from (**a**–**d**) control mice (i.e., lacking Cre) (n = 7) or from (e-h) CDX2-CreERT2:α3^flx/flx^ mice (n = 10), 9 days following initial IP injection of tamoxifen. Cryosections were stained with pre-immune serum (**a**,**e**) or anti-α3 (**b**,**f**). Paraffin sections were stained with H&E (**c**,**d**,**g**,**h**); two section planes are shown for each. Scale bars, 50 μm.

First, we determined whether *Itga3* deletion altered normal colon histology during our experimental timeframe. These experiments utilized CDX2-CreERT2:α3^flx/flx^ mice generated through the above-described intercross (Figure 1a) that did not carry the *Kras*G12D oncogene and were *Apc*^+/+^ (i.e., these mice were not susceptible to colorectal dysplasia or cancer following tamoxifen treatment). We treated CDX2-CreERT2:α3^flx/flx^ mice or control mice (i.e., CDX2-CreERT2:α3^+/+^) with tamoxifen, then isolated colon tissue 7–11 days later for histological comparison (Figure 2; 9 days pictured). IF staining of cryosections confirmed reduced α3 expression in colon from CDX2-CreERT2:α3^flx/flx^ mice (Figure 2e,f), compared with control mice (Figure 2a,b). H&E staining of paraffin sections revealed normal tissue structure in colons from both control mice (Figure 2c,d) and CreERT2:α3^flx/flx^ mice (Figure 2g,h), with parallel crypts lined with epithelial cells, including goblet cells, showing that the ablation of α3β1 did not alter the tissue structure of normal adult colons.

**Figure 3:**
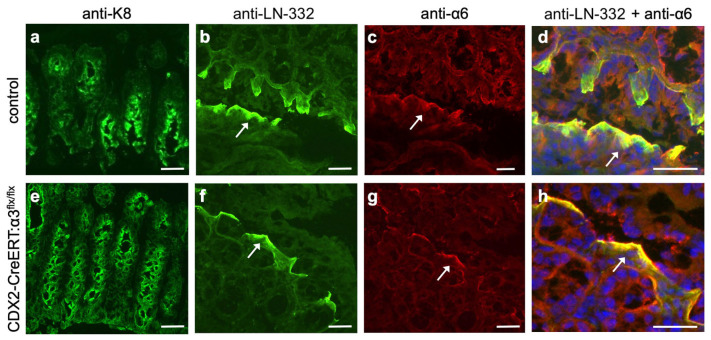
Genetic deletion of *Itga3* in colon leads to enhanced colocalization of integrin α6β4 and LN-332. Colon tissue was isolated from (**a**–**d**) control mice (i.e., lacking Cre) (n = 12), or from (**e**–**h**) CDX2-CreERT2:α3^flx/flx^ mice (n = 10), 9 days following initial IP injection of tamoxifen. Cryosections were stained with anti-K8 (**a**,**e**), or co-stained with anti-LN-332 ((**b**,**f**); green) and anti-α6 integrin ((**c**,**g**); red). (**d**,**h**) Enlargements of co-stained areas for LN-332 and α6; yellow color indicates colocalization of LN-332 and α6. Blue, DAPI; arrows point to same regions in corresponding panels. Scale bars, 50 μm.

IF revealed a similar distribution of LN-332 in colon cryosections of control mice (Figure 3a,b) or CDX2-CreERT2:α3^flx/flx^ mice (Figure 3e,f), where it localized to the basement membrane at the upper portion of the crypt, as reported previously [46]. LN-332 is a ligand for integrins α3β1 and α6β4, which are co-expressed in many epithelial tissues and carcinomas [25], including the colon epithelium and CRC [30,46]. Interestingly, the colocalization of α6 integrin with LN-332 was enhanced in the colons of tamoxifen-treated CDX2-CreERT2:α3^flx/flx^ mice (Figure 3f–h), compared with control mice (Figure 3b–d), possibly reflecting compensatory adhesion mediated by α6β4 when α3β1 is absent. Indeed, previous studies have shown that α6β4-mediated adhesion to LN-332 persists in the epidermis or cultured keratinocytes following the genetic ablation of α3β1, indicating a capacity for functional compensation between these two integrins [47,48].

### 3.3. Genetic Deletion of Itga3 Does Not Alter Dysplastic Growth in the KPC:APC Model

We next determined the effect of *Itga3* deletion in the KPC:APC model. It was reported previously that colonic tumors were detected within 25–30 days following the treatment of KPC:APC mice with tamoxifen (0.1 mg/20 gm body weight), which also caused extensive inflammation in the colon [13]. Of note, inflammation has been linked to an increased risk of CRC development, and murine models have contributed to our understanding of how inflammatory signals may contribute to CRC, as reviewed [49]. In the former study, tamoxifen-treated KPC:APC mice lived for an average of 165 days, at which point they succumbed to cachexia with rectal bleeding [13]. In contrast, we observed a substantially more rapid decline in the health of tamoxifen-treated KPC:APC mice regardless of their α3^+/+^ or α3^flx/flx^ genotype, with extensive inflammation throughout the colon (Figure S2a). We speculate that this rapid decline in health of tamoxifen-treated mice compared with the study by Maitra and coworkers [13] reflects a more severe impact of activating *Kras*G12D and/or deleting *Apc* in the mixed genetic background that resulted from crossing in the *Itga3*^flx^ allele. Of 22 total mice, four died between 10–12 days post-tamoxifen treatment due to cachexia. Of the 18 remaining mice, 16 were sacrificed between 9 and 12 days because they had lost at least 20% of body weight and/or appeared moribund, and two were sacrificed at day 14. Nine of the eighteen mice displayed bloody stools at the time of sacrifice. We did not observe differences in these phenotypes between KPC:APC:α3^+/+^ mice (n = 10) and KPC:APC:α3^flx/flx^ mice (n = 8), and there was no significant difference in weight loss between the two groups by day 9 (Figure S2b).

**Figure 4:**
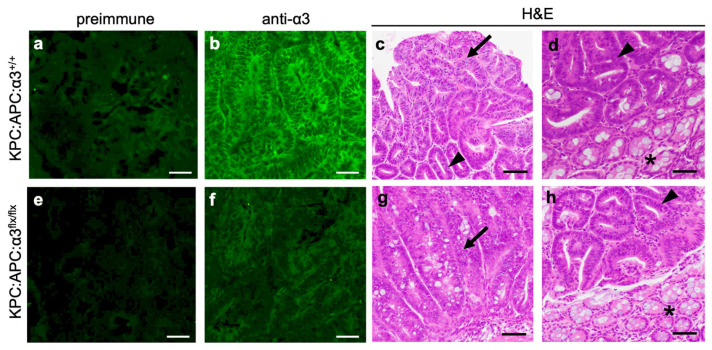
Genetic deletion of *Itga3* in the colon of KPC:APC mice does not alter colonic dysplasia. Colon tissue was isolated from tamoxifen-treated KPC:APC:α3^+/+^ mice (**a**–**d**) (n = 10), or KPC:APC:α3^flx/flx^ mice (**e**–**h**) (n = 8). Cryosections were stained with pre-immune serum (**a**,**e**) or anti-α3 (**b**,**f**) to confirm α3 deletion. Paraffin sections were stained with H&E; examples of proximal colon (**c**,**g**) and distal colon (**d**,**h**) are shown for each genotype. Arrows, examples of high-grade dysplasia; arrowheads, examples of low-grade dysplasia; asterisks, areas of normal colon. Scale bars, 50 μm.

Gross tumors were not detected in the colons of mice from either genotype by the time they died or had to be sacrificed, which precluded us from assessing the effects of α3 deletion on colon tumorigenesis. However, we were able to assess dysplasia that developed throughout the colon. IF showed that the distribution of α3 was increased in colon epithelial cells from tamoxifen-treated KPC:APC:α3^+/+^ mice, compared with normal colons (compare Figure 2b and Figure 4b). As expected, α3 staining was reduced in colons from tamoxifen-treated KPC:APC:α3^flx/flx^ mice, indicating a loss of α3β1 (Figure 4e,f). H&E-stained sections were prepared from KPC:APC:α3^+/+^ mice and KPC:APC:α3^flx/flx^ mice 9–14 days after initial tamoxifen treatment using the “Swiss-rolling” technique to maximize the histological assessment of the entire colon [50]; then, they were assessed for the presence of dysplasia by a pathologist who was blinded to genotype. Comparison of the distal or proximal colon (i.e., cecum) between KPC:APC:α3^+/+^ mice and KPC:APC:α3^flx/flx^ mice did not reveal differences in either the extent of dysplasia (Figure S3) or the proportion of dysplasia that was high-grade (Figure S4). Figure 4 shows representative images of dysplasia in the proximal or distal colon for KPC:APC:α3^+/+^ mice (Figure 4c,d) and KPC:APC:α3^flx/flx^ mice (Figure 4g,h).

**Figure 5:**
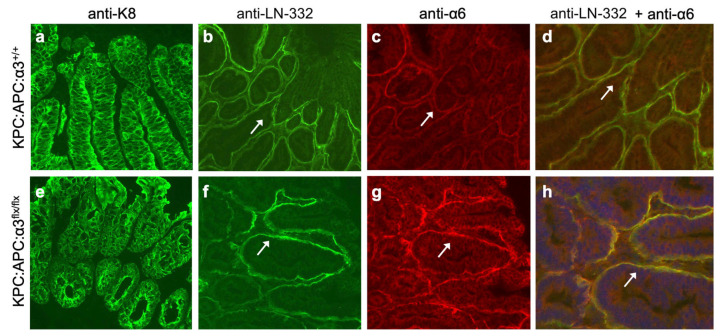
Genetic deletion of *Itga3* in dysplastic colon of KPC:APC mice leads to enhanced colocalization of integrin α6β4 and LN-332. Colon tissue was isolated from tamoxifen-treated KPC:APC:α3^+/+^ mice (**a**–**d**) (n = 10), or KPC:APC:α3^flx/flx^ mice (**e**–**h**) (n = 8). Cryosections were stained with anti-K8 (**a**,**e**), or co-stained with anti-LN-332 ((**b**,**f**); green) and anti-α6 integrin ((**c**,**g**); red). (**d**,**h**) Enlargements of co-stained areas for LN-332 and α6; yellow color indicates colocalization of LN-332 and α6. Blue, DAPI; arrows point to same regions in corresponding panels. Scale bars, 50 μm.

IF revealed abundant LN-332 adjacent to cytokeratin-8 (K8)-positive epithelial cells throughout the dysplastic colon of both tamoxifen-treated KPC:APC:α3^+/+^ mice (Figure 5a,b) and KPC:APC:α3^flx/flx^ mice (Figure 5e,f). However, α6 integrin showed increased expression and enhanced colocalization with LN-332 in tamoxifen-treated KPC:APC:α3^flx/flx^ mice (Figure 5f–h) compared with KPC:APC:α3^+/+^ mice (Figure 5b–d), possibly reflecting compensatory adhesion by α6β4, as we had observed following α3 deletion in normal, non-dysplastic colons (Figure 3).

### 3.4. RNAi-Mediated Suppression of Integrin α3β1 Reduces Motility of Colorectal Cancer Cells

**Figure 6:**
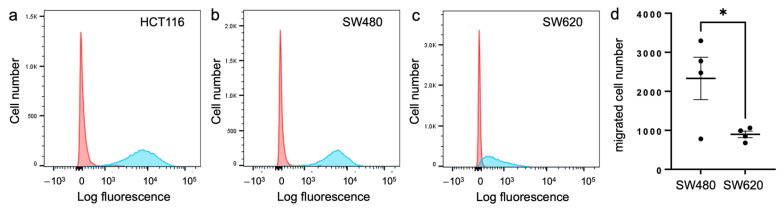
Relative expression of α3β1 in colorectal cancer cell lines. Flow cytometry with monoclonal antibody P1B5 (blue peaks) was used to compare cell-surface levels of α3β1 in three human CRC cell lines; (**a**) HCT116, (**b**) SW480 and (**c**) SW620. Red peaks, IgG control. (**d**) Migration of SW480 and SW620 cells was compared in a transwell migration assay; cells were seeded in serum-free medium with 10% serum in the lower chamber as chemoattractant. n = 4; mean ± SEM; * *p* < 0.05; two-tailed *t*-test.

The above findings revealed that α3β1 is not required for the onset of pre-cancerous dysplasia in the KPC:APC genetic model, but the rapid decline in health of these mice precluded us from determining the effect of α3 deletion on colon tumorigenesis. Since we could not use this model to determine the effect of α3β1 deficiency on colon tumorigenesis in vivo, we investigated requirements for α3β1 in the viability and motility of established human CRC cell lines. Flow cytometry with a monoclonal anti-α3 antibody revealed that α3β1 was abundant on the cell surface of HCT116 and SW480 cells, while surface expression was substantially lower on SW620 cells (Figure 6a–c). Interestingly, we observed that SW620 cells were less migratory than SW480 cells in transwell migration assays (Figure 6d), indicating that a lower expression of α3β1 is associated with reduced motility.

**Figure 7:**
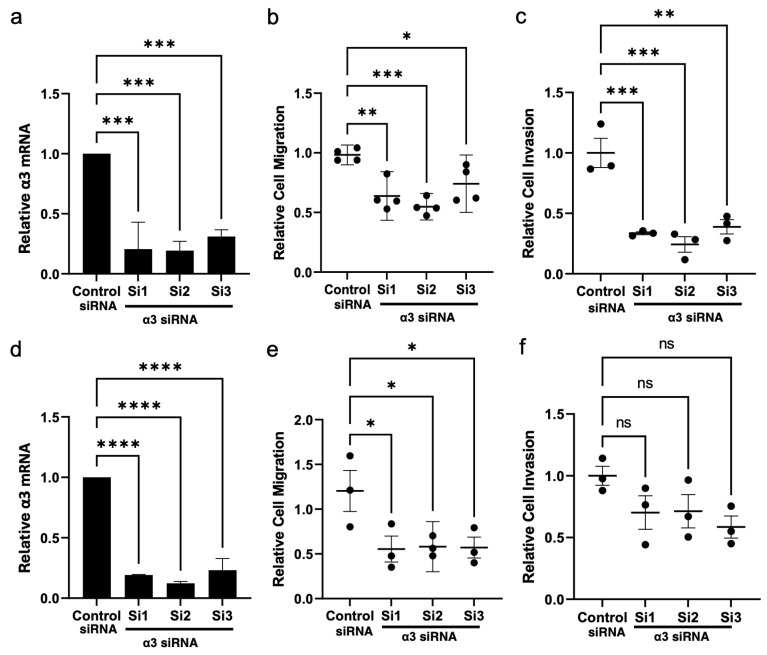
RNAi-mediated suppression of α3 reduces migration and invasion of colorectal cancer cells. SW480 cells (**a**–**c**) or HCT116 cells (**d**–**f**) were treated with three distinct siRNAs that target the α3 mRNA (Si1-3), or with a non-targeting siRNA (Control siRNA). (**a**,**d**) Knockdown of α3 mRNA was confirmed by qPCR. (**b**,**e**) Motility was compared using a transwell migration assay, as in Figure 6. (**c**,**f**) Invasion was compared using a Matrigel invasion assay; cells were seeded in 10% serum with 20% serum in the lower chamber as chemoattractant. (**a**,**d**) n = 3; (**b**–**e**) n = 3 or 4; mean ± SEM; * *p* < 0.05; ** *p* < 0.01; *** *p* < 0.001; **** *p* < 0.0001; one-way ANOVA with Dunnett multiple comparisons post-test; ns, not significant.

**Figure 8:**
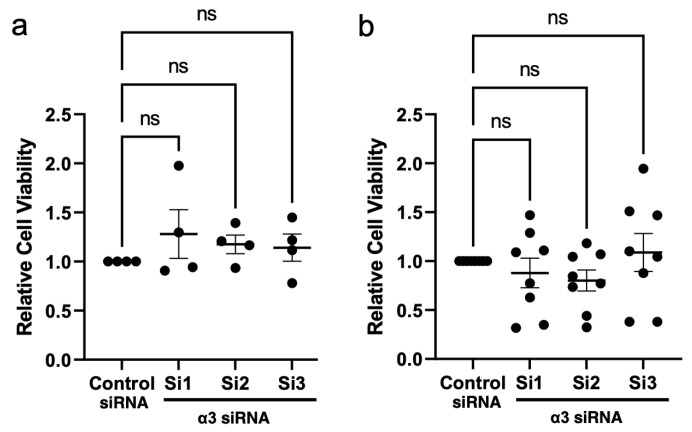
RNAi-mediated suppression of α3 has no effect on viability of colorectal cancer cells. SW480 cells (**a**) or HCT116 cells (**b**) were treated with three distinct siRNAs that target the α3 mRNA (Si1-3), or with a non-targeting siRNA (Control siRNA), then CCK8 cell viability assay was performed at day 4 and normalized to day 1. (**a**) n = 4; (**b**) n = 8; data are relative to control (set at a value of 1.0); mean ± SEM; one-way ANOVA with Dunnett multiple comparisons post-test; ns, not significant.

To directly test a requirement for α3β1 in the motility of SW480 cells or HCT116 cells, we transfected each line with three distinct siRNAs that target the *ITGA3* gene to suppress the α3 integrin subunit, or with a non-targeting siRNA as a control. Efficient knockdown of α3 was confirmed both by qPCR for the *ITGA3* mRNA (Figure 7a,d). Suppression of α3β1 in either cell line caused a significant reduction in cell motility in transwell migration assays, compared with control cells (Figure 7b,e). Moreover, the assessment of invasion using Matrigel transwell assays showed that the suppression of α3β1 in SW480 cells led to a significant reduction in their invasive potential (Figure 7c). Although the knockdown of *ITGA3* in HCT116 cells produced a similar trend towards reduced invasion, these differences did not reach statistical significance (Figure 7f). However, cell viability was not affected by α3 knockdown in either cell line (Figure 8a,b). Together, these results suggest that α3β1 is required for CRC cell motility/invasion but not for cell growth or survival.

## 4. Discussion

Understanding the mechanisms that underlie CRC pathology requires the elucidation not only of how specific driver mutations in oncogenes and tumor-suppressor genes promote disease progression through each stage but also how changes in the TME that occur at these stages support the selective survival and outgrowth of tumor cells that acquire such mutations. As a major family of cell adhesion receptors for the ECM, integrins play critical roles at all stages of cancer progression and are promising therapeutic targets [17,51,52]. There are 24 distinct integrins that can form from the limited heterodimerization of 18 α subunits and 8 β subunits, and most cells express several different integrins with distinct ligand-binding and signaling functions [15]. Importantly, some integrins are upregulated on tumor cells (at the level of expression or function) to promote tumorigenesis and metastasis, while others are downregulated due to their suppressive roles [17]. Moreover, roles for a specific integrin can differ between cancer types or even change during progression of a cancer type [17,25], which raises significant challenges in exploiting individual integrins as consistently reliable prognostic markers or effective therapeutic targets.

Roles for integrin α3β1 in cancer are complex, and whether this integrin has roles that are supportive or suppressive of tumor progression or metastasis depends on the type or subtype of cancer, as we have reviewed in detail previously [19]. Indeed, numerous preclinical studies have shown that integrin α3β1 has pro-tumorigenic/pro-metastatic roles in many cancer types, including SCC, breast cancer, glioma, melanoma, and pancreatic cancer, suggesting that it has potential value as a therapeutic target [20,21,22,23,24,25,26,27]. Moreover, a high expression of α3β1 and/or its laminin ligands has been associated with poor outcomes in SCCs and breast cancer, implicating α3β1 as a potentially useful prognostic marker [20,22,23,24,25,28,29,44,53,54,55]. However, α3β1 has also been reported to suppress tumorigenesis or metastasis in some cancer types or subtypes. For example, α3β1 signaling through the Hippo pathway suppresses cell invasion and anchorage-independent growth of prostate cancer cells [33], as well as their metastasis [56], and a suppressive role for α3β1 has been described in HER2-driven breast cancer [34]. Moreover, in some cases, α3β1 may have opposing effects at different stages of a cancer’s progression, such as the transition of epidermal tumors to SCC [35]. Collectively, the above studies indicate that α3β1 is frequently involved in advanced tumor cell motility and invasion but that such cancer-promoting roles depend on the cancer type/subtype or stage of progression. The dynamic regulation of roles for α3β1 within a cancer type could reflect changes in the availability of ECM ligands to which it binds, or of the downstream effectors through which it signals [19]. It follows that an improved understanding of the changing roles of α3β1 change during specific stages of cancer progression will inform the development of strategies to exploit this integrin as either a prognostic marker or a therapeutic target.

Unfortunately, we were unable to assess the effects of *Itga3* deletion on colon tumorigenesis in the KPC:APC mouse model of CRC due a rapid decline in their health, which led to death or euthanasia within the timeframe that tumors become detectable in this model [13]. Although this limitation precluded use of the KPC:APC mouse model to assess tumorigenesis and progression, we were able to show that the genetic deletion of *Itga3* in the colon did not alter extensive inflammation and the colonic dysplasia that forms in these mice. These observations indicate that α3β1 is not required for the onset of pre-cancerous dysplasia and that its absence does not cause a more severe phenotype in these mice.

Our observation that α6 colocalized with LN-332 to a greater extent following the deletion of α3 from colon epithelial cells, in either normal colon (i.e., of CDX2-CreERT2:α3^flx/flx^ mice) or dysplastic colon (i.e., of KPC:APC:α3^flx/flx^ mice), may reflect compensatory adhesion by α6β4 when α3β1 is absent. Indeed, the potential for overlap in the cancer-supportive roles of these two laminin-binding integrins has been reviewed extensively, and their potential to functionally compensate for one another is well-documented [22,25,52]. This interpretation is consistent with previous reports by us and others that adult mice with epidermis-specific α3 knockout, or α3-null keratinocytes, display compensatory adhesion by α6 integrins [47,48,57]. Of note, a previous study showed that α6β4 is protective of CRC at early stages [46], while other studies have demonstrated pro-cancer roles for this integrin in CRC or other carcinoma cells [58,59,60,61], possibly reflecting a switch in α6β4 function during progression. Collectively, these findings suggest that α3β1 and α6 integrins may compete with, or compensate for, one another to some extent [22,25].

Since we were unable to assess the effects of α3β1 deficiency on in vivo tumorigenesis in KPC:APC mice, we used RNAi to suppress *ITGA3* in two widely used human CRC cell lines: SW480 and HCT116 cells. We observed that knockdown of α3 in either line did not affect viability, suggesting that α3β1 is dispensable for tumor cell growth and survival. However, a knockdown of α3 caused reduced transwell motility, supporting a pro-migratory role for α3β1 as CRC cells invade from the primary tumor. This result is interesting in light of our survival analysis using the Human Protein Atlas [45], which did not reveal a correlation between higher *ITGA3* expression and poor clinical outcomes for colon cancer (Figure S1). We speculate that a requirement for α3β1 during the local invasion of the primary could be masked by its dispensability, or even inhibitory roles, at other stages.

Although the signaling pathways through which α3β1 promotes CRC cell motility remain unclear, this integrin has been linked to several downstream signaling effectors that can promote cell migration and invasion. Focal adhesion kinase (FAK) and Src family kinases are among the most widely studied integrin signaling effectors [51,62,63,64,65], and the FAK-Src axis is a common downstream effector of α3β1 signaling in normal epithelial cells and carcinoma cells [53,66,67,68]. For example, in keratinocytes, α3β1-FAK-Src signaling activates Rac1, which in turn promotes leading-edge lamellipodia and directional migration [67,69]. α3β1-FAK-Src signaling has also been linked to PI3K/Akt and STAT3 signaling pathways in a murine skin carcinogenesis model [66], and α3β1 signaling through FAK was linked to downstream effectors such as Rac1/PAK1, MAPK and JNK in a mouse model of mammary carcinoma [53], as well as to a YAP-mTOR signaling axis in a mouse model of incisor regeneration [70]. Of note, there are examples of FAK-Src signaling downstream of β1 integrins that promotes CRC migration/invasion [71,72]; whether such signaling occurs downstream of α3β1 will be elucidated in future studies.

Interestingly, we observed substantially lower α3β1 expression in SW620 cells compared with SW480 cells. The SW480 and SW620 cell lines represent a lineage of colorectal cancer progression, wherein SW480 cells were derived from an adenocarcinoma of the large intestine, while SW620 cells were established from a lymph-node metastasis of the same patient one year later [73], suggesting that α3β1 expression may be suppressed in CRC metastases. Consistently, lower *ITGA3* expression has been correlated with metastasis and poor prognosis in CRC patients, and in many cases, metastatic tumors had lower α3 expression levels than their corresponding primary tumors [38,39]. Our observations suggest that α3β1 may contribute to initial cell invasion from the primary tumor, while its expression is suppressed in CRC cells that have metastasized. We also observed that lower α3β1 expression in SW620 cells was correlated with reduced migration in transwell assays compared with SW480 cells. Interestingly, a previous study reported the opposite finding: that migration and invasion were upregulated in the metastatic SW620 cells compared with the primary SW480 cells [74]. This discrepancy could be due to the use in the latter study of transwell membranes that were coated with fibronectin, to which α3β1 does not bind but which engages other integrins, such as α5β1, that may promote CRC motility [15]. Of note, there is precedent that requirements for α3β1 can change during the development and progression of SCC, wherein α3β1 is required for both the initiation and maintained growth of epidermal tumors [27,35,75], but its absence accelerates the conversion of benign tumors to carcinomas [35]. This potential for α3β1 to switch its role from pro-tumorigenic to cancer-suppressive has obvious implications for the design of therapeutic strategies to target α3β1 at specific stages of CRC progression.

## 5. Conclusions

Our findings in human CRC cells show that the knockdown of the laminin-binding integrin α3β1 selectively inhibits motility without affecting cell proliferation, supporting a role for this integrin in early tumor cell migration/invasion that may be downregulated in metastatic cells. Moreover, findings from our genetic model indicate that α3β1 is dispensable for colonic dysplasia that precedes CRC development. Taken together, our findings indicate that requirements for α3β1 vary throughout the stages of CRC development and progression, and they suggest that therapeutic strategies to target α3β1 may be most effective if applied prior to primary tumor invasion, rather than to metastatic disease. The potential for integrin α6β4 to compensate for a loss of α3β1 in colon epithelial cells is also an important consideration when developing strategies to target the laminin-binding integrins in CRC. The rapid health decline of KPC:APC mice in our study precluded our ability to directly test the effects of α3 deletion on colon tumorigenesis and CRC progression, so translational implications in this regard remain unclear. However, future studies using alternative murine CRC models, including xenograft models [76], may be useful to validate our findings in vivo and determine roles for α3β1 at later stages of CRC.

## Data Availability

All original contributions presented in this study are included in the article/Supplementary Material; further inquiries can be directed to the corresponding author.

## Supplementary Materials

The following supporting information can be downloaded at: https://www.mdpi.com/article/10.3390/cancers17030371/s1, Figure S1: Survival analysis of *ITGA3* gene expression in different cancers; Figure S2: Inflammation of the colon and weight loss are not different in KPC:APC:α3^+/+^ mice and KPC:APC:α3^flx/flx^ mice treated with tamoxifen; Figure S3: Degree of colonic dysplasia is not different in KPC:APC:α3^+/+^ mice and KPC:APC:α3^flx/flx^ mice treated with tamoxifen; Figure S4: Degree of high-grade colonic dysplasia is not different in KPC:APC:α3^+/+^ mice and KPC:APC:α3^flx/flx^ mice treated with tamoxifen.
