# Supplementary material for: Integrin α3β1 Is Not Required for Onset of Dysplasia in Genetic Model of Colon Cancer but Promotes Motility of Colon Cancer Cells
Source: Cancers (Basel). 2025 Jan 23;17(3):371. doi: 10.3390/cancers17030371 (PMC11815772; doi:10.3390/cancers17030371)
Supplement: Supplementary file 1 [file cancers-17-00371-s001.zip › cancers-3387344-supplementary.pdf]

cancers

# Supplemental figures: Integrin α3β1 Is Not Required for Onset of Dysplasia in Genetic Model of Colon Cancer but Promotes Motility of Colon Cancer Cells.

Kathryn E. Ottaviano[1], Sita Subbaram[1,2,3], Lei Wu[1], Kiley Stahl[2], Antoinette J. Mastrangelo[2], Hwajeong Lee[4], C. Michael DiPersio[1,2*]

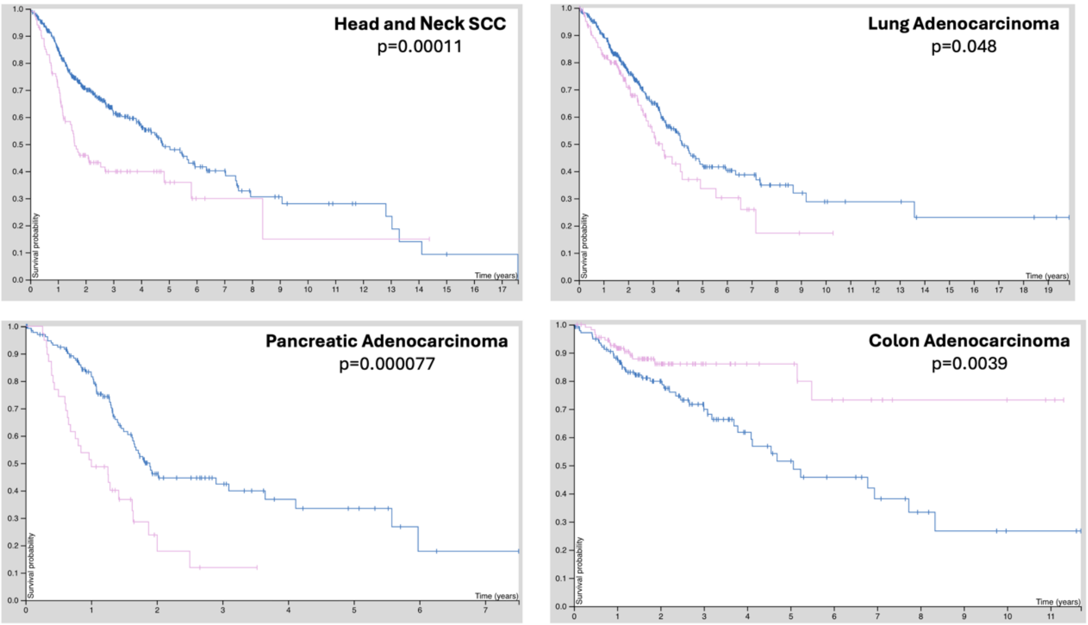


**Figure S1. Survival analysis of *ITGA3* gene expression in different cancers.** Kaplan-Meier plots show analysis of correlation between *ITGA3* mRNA expression level and patient survival for the indicated cancers. Patients were divided based on low (under cut off, *blue lines*) or high (over cut off, *pink lines*) level of *ITGA3* expression. X-axis shows time for survival (years); y-axis shows probability of survival (1.0 = 100%); log-rank P values are indicated. https://www.proteinatlas.org/ENSG00000005884-ITGA3/cancer. Image credit, Human Protein Atlas.

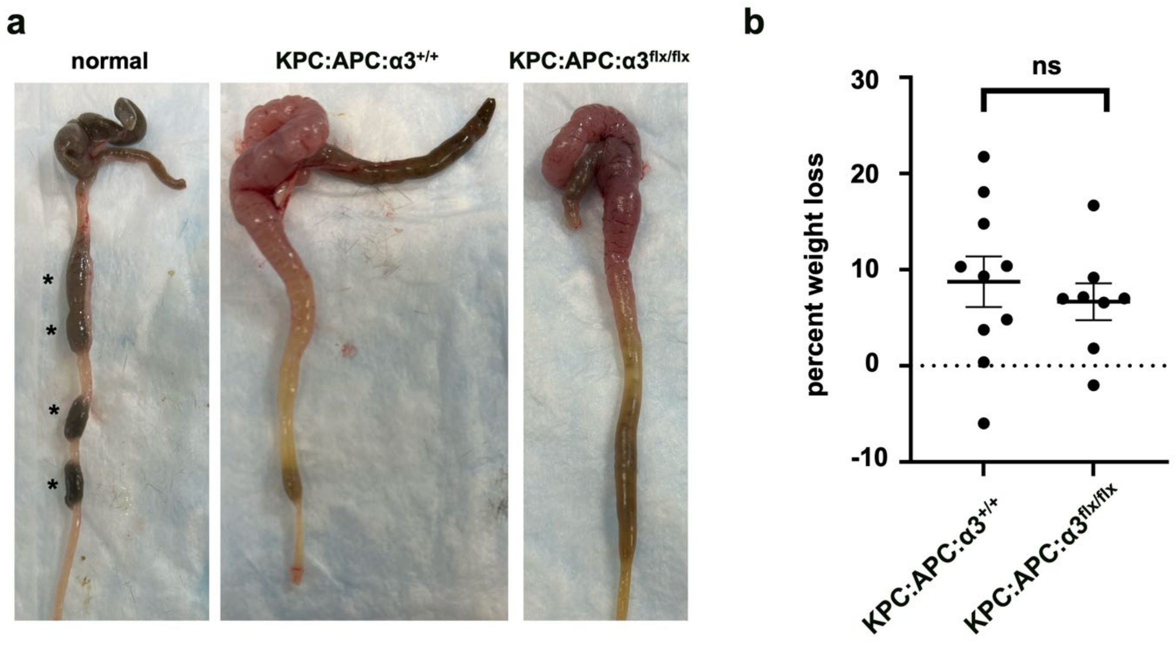


**Figure S2. Inflammation of the colon and weight loss are not different in KPC:APC:$\alpha$3$^{+/+}$ mice and KPC:APC:$\alpha$3$^{flx/flx}$ mice treated with tamoxifen.** (a) Gross anatomical appearance of colon from a normal mouse or from KPC:APC:$\alpha$3$^{+/+}$ or KPC:APC:$\alpha$3$^{flx/flx}$ mice treated with tamoxifen for 9 days. *Asterisks* indicate feces in the normal colon. (b) Graph shows percent weight loss in KPC:APC:$\alpha$3$^{+/+}$ or KPC:APC:$\alpha$3$^{flx/flx}$ mice treated with tamoxifen (see methods). Mean +/- SEM; two-tailed T-test; *ns*, not significant.

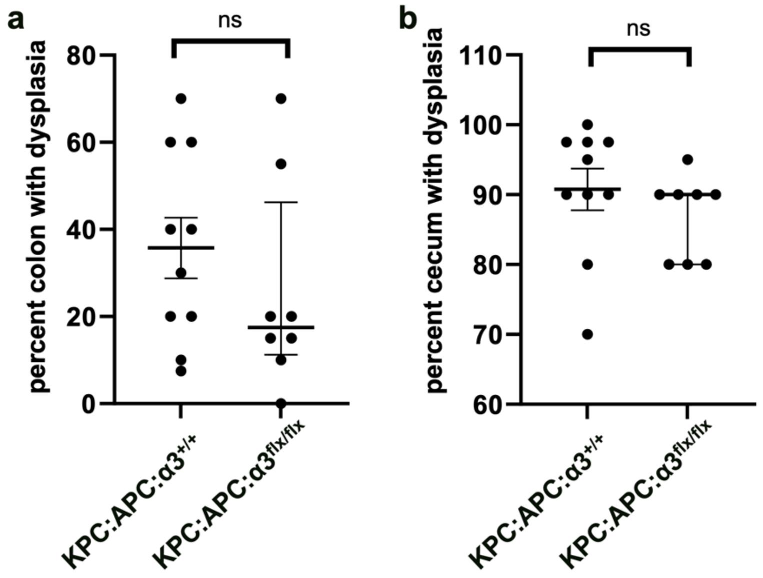


**Figure S3. Degree of colonic dysplasia is not different in KPC:APC:$\alpha$3$^{+/+}$ mice and KPC:APC:$\alpha$3$^{flx/flx}$ mice treated with tamoxifen.** Graphs show percent of dysplasia in (a) distal colon or (b) cecum of KPC:APC:$\alpha$3$^{+/+}$ or KPC:APC:$\alpha$3$^{flx/flx}$ mice treated with tamoxifen (see methods). Mean +/- SEM; two-tailed T-test; *ns*, not significant.

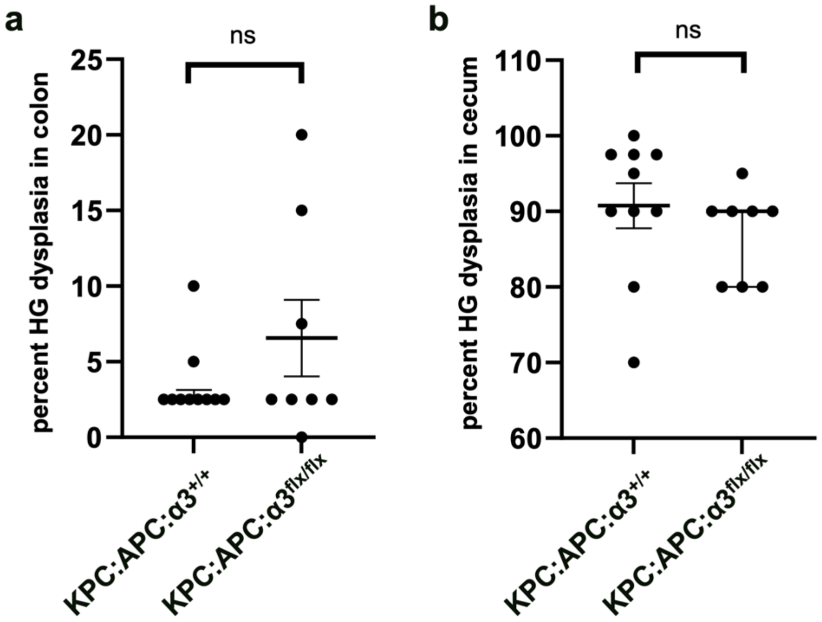


**Figure S4. Degree of high-grade colonic dysplasia is not different in KPC:APC:$\alpha 3^{+/+}$ mice and KPC:APC:$\alpha 3^{flx/flx}$ mice treated with tamoxifen.** Graphs show percent of high-grade (HG) dysplasia in (a) distal colon or (b) cecum of KPC:APC:$\alpha 3^{+/+}$ or KPC:APC:$\alpha 3^{flx/flx}$ mice treated with tamoxifen (see methods). Mean +/- SEM; two-tailed T-test; *ns*, not significant.
